# Kounis syndrome. Apropos of a clinical case

**DOI:** 10.5935/0103-507X.20200021

**Published:** 2020

**Authors:** Pedro Duarte, Joana Costa, Cátia Serena, Carla Almeida, Sandra Gouveia, César Lourenço, Humberto Costa, Clara Paiva

**Affiliations:** 1 Hospital do Divino Espírito Santo de Ponta Delgada - Ponta Delgada, Portugal.

**Keywords:** Allergy, Beta-lactams, Acute coronary syndrome, Kounis syndrome, Alergia, Beta-lactâmicas, Síndrome coronariana aguda, Síndrome de Kounis

## Abstract

Kounis syndrome, while an acute coronary syndrome, occurs in the context of a hypersensitivity reaction, allergies, or anaphylaxis and is subdivided into three types: coronary spasm in normal arteries, instability of plaques in atherosclerotic coronary arteries, and thrombosis of coronary stents. Herein, the case of a 73-year-old patient who, after administration of amoxicillin/clavulanic acid, went into cardiorespiratory arrest with evidence of ST-T segment elevation on electrocardiogram is reported. Coronarography revealed no obstructive lesions, and spontaneous resolution of electrocardiographic abnormalities was observed. A review of anamnesis with the family revealed a previous allergy to penicillin. The tryptase dosage was strongly positive. Kounis syndrome type 2 was diagnosed, and the clinical outcome was good.

## Abstract (pt)

A síndrome de Kounis, enquanto síndrome coronária aguda, ocorre em um contexto de reação de hipersensibilidade, alergia ou anafilaxia, e subdivide-se em três tipos: o espasmo coronário em artérias normais, a instabilidade de placas em artérias coronárias ateroscleróticas e a trombose de stent coronário. Apresenta-se o caso de uma doente de 73 anos que, após administração de amoxicilina/ácido clavulânico, entra em parada cardiorrespiratória, com evidência de supradesnivelamento do segmento ST-T em eletrocardiograma. Realiza coronariografia com ausência de lesões obstrutivas, verificando-se resolução espontânea das alterações eletrocardiográficas. Revisão da anamnese com a família documenta alergia prévia à penicilina. O doseamento de triptase foi fortemente positivo. Foi admitida provável síndrome de Kounis tipo 2, com boa evolução clínica posterior.

## INTRODUCTION

The first report of acute coronary syndrome in the context of allergies was described in 1950 by Pfistero in a 49-year-old man after treatment with beta-lactam antibiotics for 4 days.^(1)^ In 1991, Kounis described, for the first time, “allergic angina syndrome”, which is characterized by chest pain and allergic skin reaction, accompanied by classic clinical and laboratory findings of myocardial infarction caused by inflammatory mediators released during the allergic reaction.^(2-4)^ Kounis syndrome is subdivided into three types: type 1 results from coronary spasms in normal coronary arteries; type 2 results from spasms or plaque rupture in coronary arteries with previous atherosclerosis; and type 3 results from a hypersensitivity reaction that leads to the thrombosis of a previously implanted pharmacological stent.^(5)^

## CLINICAL CASE

**Figure 1:**
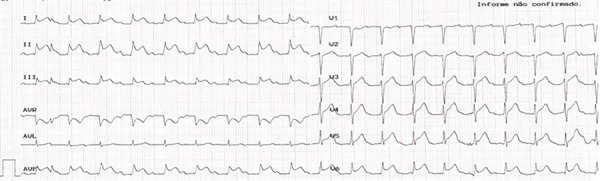
Electrocardiogram performed immediately after drug administration, with ST segment elevation in the inferior territory.

**Figure 2:**
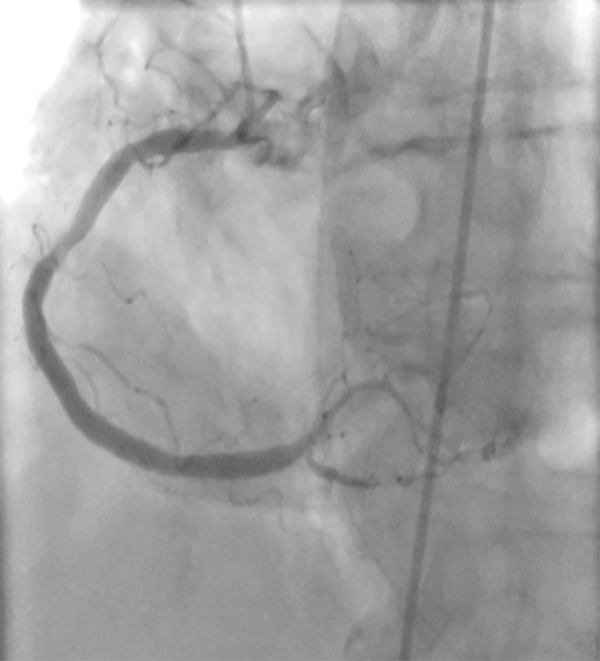
Coronarography revealed diffuse atherosclerotic disease without obstructive lesions.

The patient was a 73 years old female who was leucodermic and who had a known personal history of arterial hypertension, type 2 diabetes mellitus treated with insulin, dyslipidemia, and cerebrovascular disease. The patient denied known drug allergies. She visited the emergency department for productive cough with purulent sputum and fever (38.1°C) with 5 days of evolution. The increase in inflammatory parameters determined analytically and the condensation of the left lower lobe determined by radiology favored the diagnosis of community-acquired pneumonia. She was prescribed amoxicillin/clavulanic acid, and the first administration was by the intravenous route in the emergency department. Approximately one minute after the drug was injected, the patient exhibited a generalized skin rash and an altered state of consciousness, with peripheral oxygen saturation in room air of 67%, blood pressure 87×50 mmHg, and heart rate of 110bpm. She was treated with 2mg clemastine and 200mg hydrocortisone and unfavorably progressed to cardiorespiratory arrest, with subsequent pulse recovery after advanced life support, orotracheal intubation and mechanical ventilation. Electrocardiography showed evidence of ST segment elevation in the inferior territory (Figure 1). Urgent coronarography was performed, revealing diffuse atherosclerotic disease, with the absence of obstructive lesions (Figure 2). Spontaneous resolution of ST-T segment elevation was also observed in the hemodynamic room. The following laboratory results were obtained: troponin I peak, 2,046µg/L; total creatine kinase (CK), 647U/L; and CK-MB, 55U/L. After contact, the family mentioned a previous allergy to penicillin, which the patient was unaware of. In the first 6 hours after shock, the tryptase level was 132ng/mL (strongly positive). Considering the context of being administered amoxicillin/clavulanic acid, the patient was diagnosed with Kounis syndrome type 2. The patient remained under mechanical ventilation for 29 hours, with good subsequent clinical evolution. She was discharged with a recommendation to avoid beta-lactam antibiotics and was referred for immunotherapy.

## DISCUSSION

Allergy to beta-lactam antibiotics is the most common cause of adverse drug reactions mediated by specific immunological mechanisms.^(6)^ This is the most frequently reported drug allergy, with the prevalence varying between 5% and 10%. Recent studies show that 95% of patients with a history of penicillin allergy are not in fact allergic.^(7)^ Anaphylaxis, a measured allergic IgE reaction (type TH2), occurs through exposure to specific allergens, which induce a coordinated allergic reaction, releasing acute mediators of the inflammatory response. Mast cells are the main cells involved in allergic reactions and are present in the intima layer of the coronary arteries and atherosclerotic plaques. Given an allergic insult, they release endogenous mediators such as histamine, tryptase, leukotrienes and cytokines.^(8)^ In cases of sudden chest pain associated with symptoms of allergy or anaphylaxis, the possibility of Kounis syndrome should always be considered.^(9)^ Elevated serum tryptase levels indicate the activation of mast cells, supporting the diagnosis of anaphylaxis; however, negative values do not exclude it.^(10)^ The approach to patients with acute coronary syndrome in the context of an allergic reaction should be directed not only to the coronary event but also to the allergic reaction that induces it.^(11)^ Because vasospasm is the primary mechanism, nitrates and calcium channel blockers should be considered as first-line therapy.^(2)^ Corticosteroids are safe agents and play an important role in the treatment of allergic reactions and allergic acute coronary syndrome; however, they are associated, in some cases, with cardiac aneurysms and rupture of the ventricular wall.^(12)^ Adrenaline is the basis of anaphylaxis treatment; however, its use in Kounis syndrome can aggravate ischemia and induce coronary vasospasm and tachyarrhythmia.^(2)^ The use of acetylsalicylic acid and heparins is controversial due to their high allergenic power.^(13)^

## CONCLUSION

Kounis syndrome is most likely a common disease; however, it is underdiagnosed. It is a complex acute coronary syndrome, the pathophysiology of which is still not fully known. Timely treatment improves patient prognosis and should be directed to the hypersensitivity reaction and coronary event. More studies are needed to establish whether the use of adrenaline in these patients is safe.

## References

[1] Pfister CW, Plice SG (1950). Acute myocardial infarction during a prolonged allergic reaction to penicillin. Am Heart J.

[2] Maragkoudakis S, Hamilos M, Kallergis E, Vardas P (2013). Type 2 Kounis syndrome in an allergic woman: An uncommon presentation of acute coronary syndrome. J Cardiol Cases.

[3] Biteker M (2010). Current understanding of Kounis syndrome. Expert Rev Clin Immunol.

[4] Kounis NG (2006). Kounis syndrome (allergic angina and allergic myocardial infarction): a natural paradigm?. Int J Cardiol.

[5] Akyel A, Murat SN, Cay S, Kurtul A, Ocek AH, Cankurt T (2012). Late drug eluting stent thrombosis due to acemetacine: type III Kounis syndrome: Kounis syndrome due to acemetacine. Int J Cardiol.

[6] Torres MJ, Blanca M, Fernandez J, Romano A, Weck A, Aberer W, Brockow K, Pichler WJ, Demoly P, EAACI Interest Group on Drug Hypersensitivity (2003). Diagnosis of immediate allergic reactions to beta-lactam antibiotics. Allergy.

[7] Solensky R (2012). Allergy to ß-lactam antibiotics. J Allergy Clin Immunol.

[8] Ariza A, Mayorga C, Fernandez TD, Barbero N, Martín-Serrano A, Pérez-Sala D (2015). Hypersensitivity reactions to ß-lactams: relevance of hapten-protein conjugates. J Investig Allergol Clin Immunol.

[9] Ridella M, Bagdure S, Nugent K, Cevik C (2009). Kounis syndrome following beta-lactam antibiotic use: review of literature. Inflamm Allergy Drug Targets.

[10] República Portuguesa, Serviço Nacional de Saúde Norma nº 014/2012 de 16/12/2012 atualizada a 18/12/2014. Anafilaxia: Abordagem clínica.

[11] Kounis NG (2016). Kounis syndrome: an update on epidemiology, pathogenesis, diagnosis and therapeutic management. Clin Chem Lab Med.

[12] Gázquez V, Dalmau G, Gaig P, Gómez C, Navarro S, Mercé J (2010). Kounis syndrome: report of 5 cases. J Investig Allergol Clin Immunol.

[13] Kounis NG, Kouni SN, Koutsojannis CM (2005). Myocardial infarction after aspirin treatment, and Kounis syndrome. J R Soc Med.

